# Phenotypic Characterization of Chinese Rhesus Macaque Plasmablasts for Cloning Antigen-Specific Monoclonal Antibodies

**DOI:** 10.3389/fimmu.2019.02426

**Published:** 2019-10-11

**Authors:** Fan Zhang, Longyu Wang, Xuefeng Niu, Jiashun Li, Jia Luo, Yupeng Feng, Yanjia Yang, Ping He, Wenxia Fan, Renshan Liang, Zhiqiang Zheng, Weiqi Pan, Chufang Li, Yee Joo Tan, Haijian Yu, Ling Chen, Pingchao Li

**Affiliations:** ^1^State Key Laboratory of Respiratory Disease, Guangdong Laboratory of Computational Biomedicine, Guangzhou Institutes of Biomedicine and Health, Chinese Academy of Sciences, Guangzhou, China; ^2^Institute of Physical Science and Information Technology, Anhui University, Hefei, China; ^3^State Key Laboratory of Respiratory Disease, First Affiliated Hospital of Guangzhou Medical University, Guangzhou, China; ^4^Department of Respiratory Medicine, Huadu People's Hospital, Guangzhou, China; ^5^Department of Microbiology and Immunology, Yong Loo Lin School of Medicine, National University Health System, National University of Singapore, Singapore, Singapore; ^6^Institute of Molecular and Cell Biology, A^*^STAR (Agency for Science, Technology and Research), Singapore, Singapore

**Keywords:** plasmablast, B cell, Chinese rhesus macaques, monoclonal antibodies, influenza virus, vaccination

## Abstract

Rhesus macaques (*Macaca mulatta*) are used as a human-relevant animal species for the evaluation of vaccines and as a source for cloning monoclonal antibodies (mAbs) that are highly similar to human-derived antibodies. Although antibody-secreting plasmablasts in humans are well-defined and can be easily isolated for mAb cloning, it remains unclear whether the same phenotypic markers could be applied for isolating antibody-secreting plasmablasts from Chinese rhesus macaques. In this study, we evaluated a series of cell surface and intracellular markers and identified the phenotypic markers of plasmablasts in Chinese rhesus macaques as CD3^−^CD14^−^CD56^−^CD19^−^CD27^−^CD20^−/low^CD80^+^HLA-DR^+^CD95^+^. After influenza virus vaccination, the plasmablasts in peripheral blood mononuclear cells (PBMCs) increased transiently, peaked at day 4–7 after booster vaccination and returned to nearly undetectable levels by day 14. Antigen-specific enzyme-linked immunosorbent spot (ELISPOT) assays confirmed that the majority of the plasmablasts could produce influenza virus-specific antibodies. These plasmablasts showed transcriptional characteristics similar to those of human plasmablasts. Using single-cell PCR for immunoglobulin heavy and light chains, most mAbs cloned from the CD3^−^CD14^−^CD56^−^CD19^−^CD27^−^CD20^−/low^CD80^+^HLA-DR^+^CD95^+^ plasmablasts after vaccination exhibited specific binding to influenza virus. This study defined the phenotypic markers for isolating antibody-secreting plasmablasts from Chinese rhesus macaques, which has implications for efficient cloning of mAbs and for the evaluation of plasmablast response after vaccination or infection in Chinese rhesus macaques.

## Introduction

Non-human primates (NHPs) are widely used as a model for the evaluation of human vaccines and for cloning monoclonal antibodies (mAbs) against influenza virus, HIV, Ebola virus, and other pathogens (1–6). Antibody-secreting plasmablasts are a good source for cloning antigen-specific mAbs after vaccination or infection with a pathogen (7–10). In humans, plasmablasts have been well-characterized and defined as CD3^−^CD19^+^CD20^−/low^CD27^hi^CD38^hi^ cells (7, 11). In humans, plasmablasts peak around 7 days after influenza virus vaccination (7, 12), and influenza virus-specific antibody-secreting cells account for up to 6% of all B cells (7). However, the same phenotypic markers may not be useful for identifying rhesus macaque (*Macaca mulatta*) plasmablasts. Previous studies investigating how to identify rhesus macaque plasmablasts and their kinetics after vaccination have mostly been focused on Indian rhesus macaques (8, 9). Moreover, the phenotypic markers used to characterize antibody-secreting cells in rhesus macaques have been inconsistent among different laboratories. In Indian rhesus macaques, one paper reported that the plasmablasts were CD3^−^CD16^−^CD20^−/low^HLA-DR^+^CD14^−^CD11c^−^CD123^−^CD80^+^ cells, and the magnitude of the simian immunodeficiency virus (SIV) gp140-specific plasmablast response following booster vaccination was significantly higher at day 4 than at day 7 (8). Another paper suggested that the Indian rhesus macaque plasmablasts were CD3^−^CD19^low/+^CD20^−/low^sIgG^−^CD38^+^CD27^−/+^ cells, and the plasmablasts peaked at day 7 after vaccination with live attenuated dengue viruses (9). It is important to note that in these two reports, a different set of cell surface markers were used. The research on the phenotypic markers of plasmablasts from Chinese rhesus macaques is even more lacking. One study focused on antibody-secreting plasma cells in bone marrow from Chinese rhesus macaques (13), but there have been no further studies on plasmablasts in the peripheral blood. In recent years, Chinese rhesus macaques have been increasingly used in biomedical research, possibly due to the restricted supply of Indian rhesus macaques, as India banned the exportation of rhesus macaques, while the exportation of Chinese rhesus macaques has increased in recent decades (14, 15).

The divergence between Indian and Chinese rhesus macaques has been estimated to have occurred about 162,000 years ago (16). A study reported that an HIV vaccine-induced specific T cell responses and lymphoproliferative responses in Chinese rhesus macaques were significantly weaker than those in Indian rhesus macaques, but antibody responses were stronger in Chinese rhesus macaques (17). Therefore, the immune response to an antigen may not be identical between Indian and Chinese rhesus macaques. At present, the phenotypic markers and kinetic responses of plasmablasts after vaccination in Chinese rhesus macaques remain unclear. Therefore, there is a need to define suitable phenotypic markers for identification and isolation of plasmablasts of Chinese rhesus macaques, which should greatly facilitate vaccine and antibody research using Chinese rhesus macaques.

In this study, we characterized the phenotypic markers of plasmablasts from the peripheral blood of Chinese rhesus macaques. Using influenza virus as a model antigen, we also demonstrated that antigen-specific mAbs could be efficiently cloned using single-cell PCR from flow cytometry sorted single plasmablasts after vaccination.

## Materials and Methods

### Vaccination of Chinese Rhesus Macaques and Human Volunteers

The Chinese rhesus macaques were housed at the experimental animal center of Guangzhou Institutes of Biomedicine and Health (GIBH), Chinese Academy of Sciences. The animals were cared for in conformance with the guidelines of the US National Institutes of Health (NIH) Guide for the Care and Use of Laboratory Animals and the policies and procedures of GIBH. The protocol was approved by our Institutional Animal Care and Use Committee. The eight Chinese rhesus macaques used in this study (no. R080066, R063585, R080040, R080039, R061217, R052419, R071510, and R081210) were 3–5 kg and 6–9 years old. They were immunized via intramuscular injection of 15 μg of inactivated recombinant viruses with one of these vaccines: (1) 2015–16 trivalent seasonal influenza vaccine (A/H1N1/California/2009, A/H3N2/Switzerland/2013, B/Phuket/2013), (2) A/H5N6/Guangzhou/2014, and (3) A/H7N9/Anhui/2013 in A/Puerto Rico/8/34 (PR8) background in 1 ml. Five healthy volunteers received the 2015–2016 trivalent seasonal influenza vaccine via intramuscular injection (Aleph Biomedical Co. Ltd., Dalian, China) after signed informed consent was obtained.

### Isolation of Peripheral Blood Mononuclear Cells (PBMCs)

Fresh blood samples from the Chinese rhesus macaques or humans were collected in Plasma Preparation Tube (PPT) tubes containing K_2_EDTA (BD Biosciences) at different time points after vaccination. PBMCs were isolated with OptiPrep lymphocyte separation solution (Axis Shield Poc As, Oslo, Norway) by following the manufacturer's instructions. Briefly, the tubes were centrifuged at 1,000 g for 30 min at room temperature, with slow acceleration and deceleration, and PBMCs were collected. The remaining red blood cells among the PBMCs were lysed by incubation with ammonium-chloride-potassium (ACK) lysis buffer for 5 min at 4°C and the PMBCs were then washed twice with Roswell Park Memorial Institute (RPMI)-1640. For enzyme-linked immunosorbent spot (ELISPOT) assay, the cells were suspended in R10 medium (RPMI-1640, 0.05 mM 2-mercaptoethanol, 1 mM sodium pyruvate, 2 mM L-glutamate, 10 mM 4-(2-hydroxyethyl)-1-piperazineethanesulfonic acid [HEPES], and 10% fetal bovine serum) supplemented with penicillin/streptomycin (1 × ). For cell staining, the cells were suspended in 2% fetal bovine serum (FBS) in phosphate-buffered saline (PBS).

### Antigen-Specific or IgG-Secreting Cell ELISPOT Assays

B cell ELISPOT assays were performed as previously described (18), with minor modifications. Briefly, MultiScreen 96-well filter plates (Merck Millipore, Darmstadt, Germany) were coated with 10 μg/ml inactivated influenza virus or anti-human IgG Fc antibody (Sigma) overnight at 4°C for enumeration of influenza virus-specific antibody-secreting cells or total IgG-secreting cells, respectively. The wells were washed with PBS and blocked with R10 medium for 2 h at 37°C. Whole PBMCs or PBMC-derived fluorescence-activated cell sorting (FACS)-sorted cells (described in the section below) were plated and incubated overnight in a 5% CO_2_ incubator at 37°C. The plates were washed with PBS-Tween 20 (PBST), followed by incubation with biotinylated anti-human IgG and horseradish peroxidase (HRP)-conjugated streptavidin (BD Pharmigen). Spots were developed using 3-amino-9-ethylcarbazole (AEC) substrate (BD Pharmigen). To stop the reaction, the plates were washed with water. Spots of antibody-secreting cells were counted using an ELISPOT reader (Bioreader 4000, BIOSYS, Germany). The number of spots is reported as the number of antigen-specific cells or IgG antibody-secreting cells per million cells.

### Analytical Flow Cytometry and Cell Sorting

For analytical flow cytometry, PBMCs were surface stained with fluorescein-labeled or biotin-labeled antibodies (Panel 1: CD3-Pacific Blue, CD14-phycoerythrin [PE], CD56-PE, CD19-PE-Cyanine7 [Cy7], CD20-PE-CF594, CD27-peridinin chlorophyll protein [PerCP]-Cy5.5, CD80-allophycocyanin [APC]-Cy7, human leukocyte antigen DR isotype [HLA-DR]-APC, CD95-fluorescein isothiocyanate [FITC], and IgG-biotin; Panel 2: CD3-PE, CD14-PE, CD56-PE, CD19-Pacific Blue, CD20-PE-CF594, CD27-PerCP-Cy5.5, CD80-Alexa Fluor 700, HLA-DR-APC, and CD95-FITC) for 30 min in the dark at 4°C (Supplementary Table 1). This was followed by the addition of V500-coupled streptavidin for Panel 1. The PBMCs were then washed with 2% FBS in PBS and resuspended in 2% FBS in PBS. In some experiments, intracellular staining was conducted after surface staining. The cells were permeabilized and underwent intracellular staining using a Foxp3/transcription factor staining buffer set (eBioscience). The stained cells were run on a BD LSRFortessa cell analyzer (BD Biosciences). All data were analyzed using FlowJo software (Tree Star, Ashland, OR, USA).

For sorting the macaque plasmablasts, fresh isolated PBMCs were surface stained with a cocktail of fluorescein-labeled antibodies (CD3-Pacific Blue, CD14-PE, CD56-PE, CD19-PE-Cy7, CD20-PE-CF594, CD27-PE, CD27-PerCP-Cy5.5, CD80-APC-Cy7, HLA-DR-APC, and CD95-FITC) in the dark at 4°C (Supplementary Table 1). These cells were sorted as bulk cells for subsequent functional ELISPOT assays (described in the section above). To identify plasmablasts from Chinese rhesus macaques, a sequential flow cytometry gating strategy was developed to enrich for plasmablasts: (1) extended lymphocyte gate that included plasmablasts was made on the forward scatter-area (FSC-A)/side scatter-area (SSC-A); (2) singlet lymphocytes were identified using the SSC-A/side scatter-width (SSC-W) and FSC-A/forward scatter-width (FSC-W); (3) a lineage-negative gate (CD3^−^CD14^−^CD56^−^) to exclude granulocytes, CD3^+^ T cells, CD14^+^ monocytes, and CD56^+^ NK cells.

### Quantitative Real-Time (qRT)-PCR for Assessing the Expression of IgG and Transcription Factors

Total RNA was extracted from the sorted cells using an extraction kit (Axygen, NY, USA) and reverse transcribed into cDNA using an iScript RT kit (Bio-Rad, Hercules, CA, USA). The cDNA then served as templates for qRT-PCR and were amplified using a Bio-Rad CFX96 Real-time PCR Detection System (Bio-Rad) with QuantiFast SYBR Green PCR Master Mix (Qiagen, Hilden, Germany). Cycle threshold [C(t)] values and melting curves were analyzed with CFX Manager 3.1 (Bio-Rad). The expression of IgG and the following 11 transcription factors was assessed: Ki67, paired box 5 (Pax-5), B-cell CLL/lymphoma 6 (Bcl-6), basic leucine zipper transcription factor 2 (BACH2), zinc finger and BTB domain-containing 20 (ZBTB20), interferon regulatory factor 4 (IRF-4), X-box-binding protein 1 (XBP-1), B lymphocyte-induced maturation protein 1 (Blimp-1), T-box transcription factor (T-bet), myeloid cell leukemia 1 (MCL1), POU class 2-associating factor 1 (POU2AF1/OBF1). The expression levels were determined by comparison with the levels of beta-actin (Supplementary Table 2). The data are reported as the mean values of at least three experiments.

### Single-Cell PCR for Cloning mAbs

Reverse transcription and single-cell IgG cloning were carried out as previously described (19), with a small modification. Briefly, single plasmablasts were sorted directly into 96-well PCR plates containing 20 μl lysis buffer. The lysis buffer was composed of 40 U RNaseOUT, 6.25 mM dithiothreitol (DTT), 5 μl 5 × First-Strand buffer (250 mM Tris-HCl, pH 8.3, 375 mM KCl and 15 mM MgCl_2_), and 0.0625 μl Igepal (Sigma). Next, 150 ng random hexamers (Takara), 0.4 mM dNTPs (Fermentas), and 50 U Superscript III (Invitrogen) was added, with a total volume of 5 μl. cDNA synthesis was performed for 50 min at 50°C, 5 min at 85°C, and cooling at 4°C. The cDNA was stored at −80°C. Antibody genes were amplified from the cDNA by nested PCR, using previously described IgG heavy and light chain-specific primers (20), with some modifications (Supplementary Tables 3, 4). Briefly, all PCR reactions were performed in 96-well plates in a volume of 25 μl per well, which contained 2.5 μl cDNA, 2.5 μl 10 × PCR buffer (Qiagen), 0.5 μl 10 mM dNTP (Fermentas), 1 U HotStar Taq Plus (Qiagen), and 0.5 μM of each primer mixture. The PCR program was initiated by incubation at 94°C for 5 min, followed by 50 cycles at 94°C, for 30 s, 55°C (1st) or 60°C (2nd), for 30 s, and 70°C for 1 min, and a final elongation step at 70°C for 7 min before cooling to 4°C. The 2nd round PCR products were evaluated on 2% agarose gels, purified using QIAquick spin columns (Qiagen) and sequenced using 2nd round PCR reverse primers.

Cloning was carried out using an In-Fusion HD Cloning kit (cat. no. 638909; Clontech). Briefly, 50 ng purified PCR products were mixed with 2 ml In-Fusion HD enzyme premix and 100 ng linearized vector. Water was added to create a total volume of 10 ml. The reaction mixture was incubated for 15 min at 50°C and then placed on ice. About 4 ml of the product was used to transform TOP10 Chemically Competent *E. coli* (TransGen Biotech, Beijing, China). Five colonies for each single cell PCR were picked for sequencing confirmation. All five sequences were identical. This has been rectified in Material and Methods.

### Antibody Sequence Analysis and Expression

Regarding the single sorted plasmablasts, to identify the V(D)J family gene usage and their complementarity-determining region 3 (CDR3) lengths, V domain sequences were analyzed for the immunoglobulin (Ig) heavy chain variable gene cluster (IGHV), Ig kappa light chain variable gene cluster (IGKV), and Ig lambda light chain variable gene cluster (IGLV) using IMGT/V-QUEST (http://www.imgt.org) and the international ImMunoGeneTics information system. Heavy- and light-chain plasmids were co-transfected into 293T cells for transient expression. The supernatants were harvested at 3–5 days after transfection. Antibodies were purified with Protein A beads (cat. no. CA-PRI-0100; Repligen) according to the manufacturer's instructions.

### Antibody-Binding Enzyme-Linked Immunosorbent Assay (ELISA)

The plasmablast-derived mAbs were evaluated using ELISA. First, 1 μg/ml inactivated influenza virus (H3N2/Switzerland/2013) was coated onto 96-well plates at 4°C overnight. Serial dilutions of the supernatants of the 293T cells or purified antibodies in PBS were incubated in the wells for 2 h. The plates were washed and HRP-conjugated goat anti-human IgG was added. The reaction was developed with 3,3′,5,5′-tetramethylbenzidine (TMB) substrate according to the manufacturer's instructions (Merck Millipore). Optical density (OD) at 450 nm was measured. The minimum mAb concentration indicating antigen-specific binding was defined as an OD value ≥2-fold the OD value of the negative control.

### FACS Titration of mAb Binding to Influenza Virus Infected Cells

FACS titration of mAbs binding to influenza virus infected cells were performed as previously described with minor modifications (21). Briefly, Madin-Darby canine kidney (MDCK) cells grown in 6-well plate were inoculated with influenza virus H3N2/Switzerland/2013 for 2 h and then incubated in DMEM culture medium containing 0.3% bovine serum albumin (BSA) and N-tosyl-L-phenylalanyl chloromethyl ketone (TPCK)-trypsin (1 μg/ml, Sigma) at 37°C for 24 h. Infected cells were collected and permeabilized using a fixation/permeabilization solution kit (BD). Cells were intracellular stained by primary antibody (puritified plasmablast-derived mAbs) at different concentration and fluorescein-labeled secondary antibody (IgG-APC-H7). The stained cells were run on a flow cell analyzer.

### Data Analysis

Flow cytometric data were analyzed using FlowJo v10 software (Tree Star, Inc., Ashland, OR, USA). Statistical analyses and the construction of graphs were conducted using GraphPrism 5.01 (GraphPad Software Inc., La Jolla, CA, USA). Two-tailed *p*-values were calculated, and differences were considered significant when *p* < 0.05.

## Results

### The Cell Surface Markers for Human Plasmablasts Could Not Be Used for Identifying Chinese Rhesus Macaque Plasmablasts

**Figure 1:**
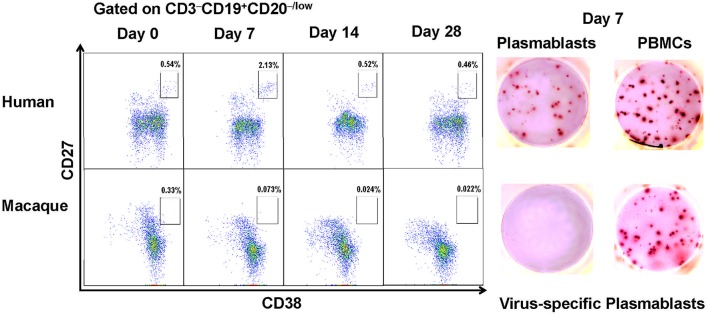
Antibody-secreting plasmablasts from Chinese rhesus macaques are phenotypically distinct from human antibody-secreting plasmablasts. Plasmablasts in PBMCs were investigated using flow cytometry. The frequencies of plasmablasts, using human plasmablast gate (CD3^−^CD19^+^CD20^−/low^CD27^hi^CD38^hi^), are shown for a representative human donor and a Chinese rhesus macaque at 0, 7, 14, 28 days after vaccination. Representative ELISPOT results showing reactivity to influenza viruses H1N1/California/2009 and H3N2/Switzerland/2013 at day 7 after vaccination. Each well contained 400 CD3^−^CD19^+^CD20^−/low^CD27^hi^CD38^hi^ cells or 2 × 10^5^ PBMCs (*n* = 4). The experiment has repeated a minimum of three times.

In humans, the cell surface markers for identifying plasmablasts have been established as CD3^−^CD19^+^CD20^−/low^CD27^hi^CD38^hi^ (7, 11). Based on these cell surface markers, we observed an increase in influenza virus-specific plasmablasts after vaccination with a seasonal influenza virus vaccine in human volunteers. CD3^−^CD19^+^CD20^−/low^CD27^hi^CD38^hi^ cells peaked at approximately 7 days and decreased by 14 days after vaccination (Figure 1). The secretion of influenza virus-specific antibodies by these cells was confirmed by B cell ELISPOT against influenza viruses. We first assessed the cross-reactivity of a variety of anti-human antibodies to ensure that they recognize the same proteins from Chinese rhesus macaques (Supplementary Table 1).

When the same cell surface markers were used for sorting cells from PBMCs obtained from Chinese rhesus macaques vaccinated with influenza viruses, few CD3^−^CD19^+^CD20^−/low^CD27^hi^CD38^hi^ cells were observed to secrete influenza virus-specific antibodies (Figure 1). Therefore, the cell surface markers for identifying human plasmablasts are not useful for identifying plasmablasts from Chinese rhesus macaques. It is thus necessary to define appropriate markers for identifying plasmablasts from Chinese rhesus macaques.

### Plasmablasts Induced After Vaccination Were Primarily CD19 Negative

**Figure 2:**
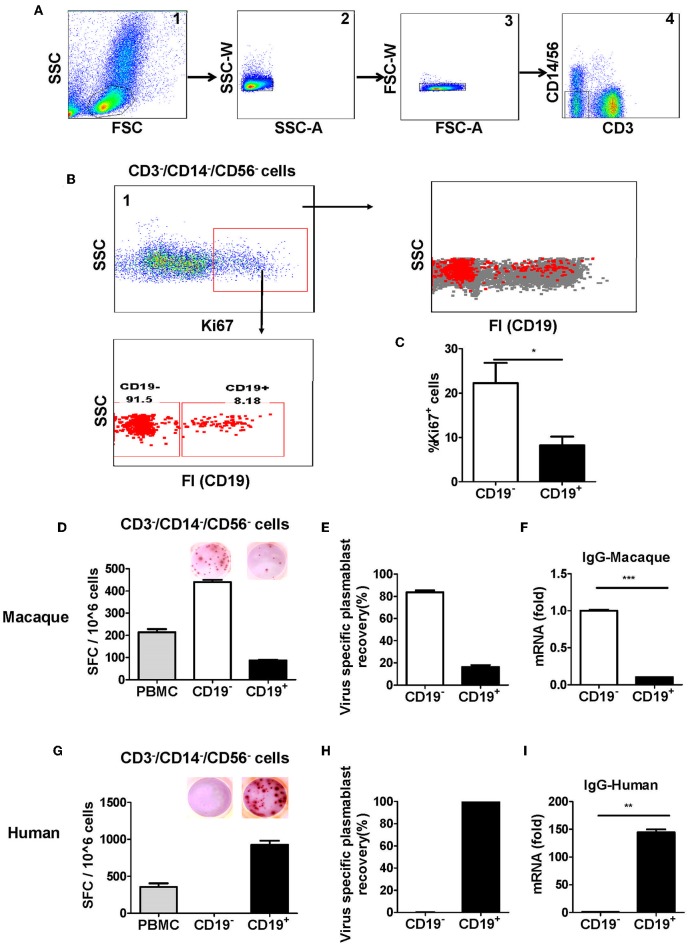
Antibody-secreting plasmablasts from Chinese rhesus macaques are primarily Ki67^+^CD19^−^ cells. A gating strategy was designed to exclude lineage cells (CD3^+^ T cells, CD14^+^ monocytes, and CD56^+^ NK cells) and the remaining cells were analyzed for intracellular Ki67. **(A)** Representative flow cytometry analysis showing the following sequential gating strategy to enrich possible plasmablasts: (1) extended lymphocyte gate that included plasmablasts; (2, 3) singlets; (4) CD3^−^CD14^−^CD56^−^ cells. **(B)** Representative flow cytometry analysis showing CD19 expression on the surface of intracellular Ki67^+^ cells. **(C)** Flow cytometry analysis showing the percentage of intracellular Ki67^+^ cells among CD19^−^ and CD19^+^ cells (*n* = 4, Mean ± SEM). PBMCs from rhesus macaques **(D,F)** (*n* = 4) and human volunteers **(G,I)** (*n* = 3) were sorted from among the CD3^−^CD14^−^CD56^−^ cells based on positive or negative CD19 expression. They were subsequently subjected to influenza A virus-specific ELISPOT assay and qRT-PCR to assess the mRNA expression levels of IgG. Data points represent the plasmablast numbers observed per million PBMCs or other cell subsets. Values represent the mean percentage of total Ig-secreting plasmablasts recovered from each cell population in Chinese rhesus macaques (Mean ± SEM) **(E)** (*n* = 4) and human volunteers **(H)** (*n* = 3). The experiment has repeated a minimum of three times. FSC, forward scatter; SSC, side scatter; FI, fluorescence intensity; SFC, spot-forming cells. *p < 0.05; **p < 0.01; ***p < 0.001.

To identify plasmablasts from Chinese rhesus macaques, we vaccinated Chinese rhesus macaques intramuscularly with influenza viruses. PBMCs were collected at 0, 4, 7, 14, and 28 days after vaccination and analyzed by FACS using a series of antibodies against cell surface markers. It is known that human plasmablasts generated after vaccination are positive for the proliferation marker Ki67 (7). Therefore, we verified the expression of the intracellular marker Ki67 in the antibody-secreting plasmablasts (Supplementary Figure 1A). Previous reports on plasmablasts from Indian rhesus macaques were controversial, as one paper reported that plasmablasts producing antigen-specific antibodies are CD19^−^ (8), whereas another paper reported that antigen-specific antibody-secreting cells are CD19^+^ (9). To clarify whether CD19 is expressed on antibody-secreting plasmablasts from Chinese rhesus macaques, we designed a flow cytometry strategy involving a singlet lymphocyte gate (which was extended to include B cells and plasmablasts) and a lineage-negative gate (CD3^−^CD14^−^CD56^−^) to exclude granulocytes, CD3^+^ T cells, CD14^+^ monocytes, and CD56^+^ NK cells (Figure 2A). After the remaining cells were stained to detect the proliferation marker Ki67, we found that >90% of the Ki67^+^ cells were CD19^−^ (Figure 2B). Thus, compared with CD19^+^ cells, CD19^−^ cells had a higher level of Ki67^+^ (Figure 2C), suggesting that these CD19^−^ cells were recently proliferating cells and are more likely to be the antibody-secreting cells.

To confirm the findings from the Ki67 analysis, CD19^−^, and CD19^+^ cells from PBMCs obtained at 7 days after vaccination were sorted from among the cells in the lineage-negative gate (CD3^−^CD14^−^CD56^−^) and tested for antibody secretion using an antigen-specific ELISPOT assay for influenza viruses. The result showed that >85% of influenza virus-specific antibody-secreting cells were in the CD19^−^ cell population, whereas <15% of them were in the CD19^+^ cell population (Figures 2D,E). Thus, most of the antigen-specific antibody-secreting cells were CD19^−^ (Figures 2D,E). To further confirm this phenotype, PBMCs were sorted from the CD3^−^CD14^−^CD56^−^ cells based on positive or negative CD19 expression, and the expression of IgG mRNA in CD19^+^ and CD19^−^ cells was determined by qRT-PCR. Compared to the CD19^+^ cells, the CD19^−^ cells had a higher level of IgG expression (Figure 2F). This result demonstrated that, after vaccination, the antigen-specific plasmablasts from Chinese rhesus macaques are phenotypically distinct from their human counterparts, and they are primarily CD19^−^ cells (Figures 2G–I).

### Plasmablasts Induced After Vaccination Were Primarily CD27 Negative

**Figure 3:**
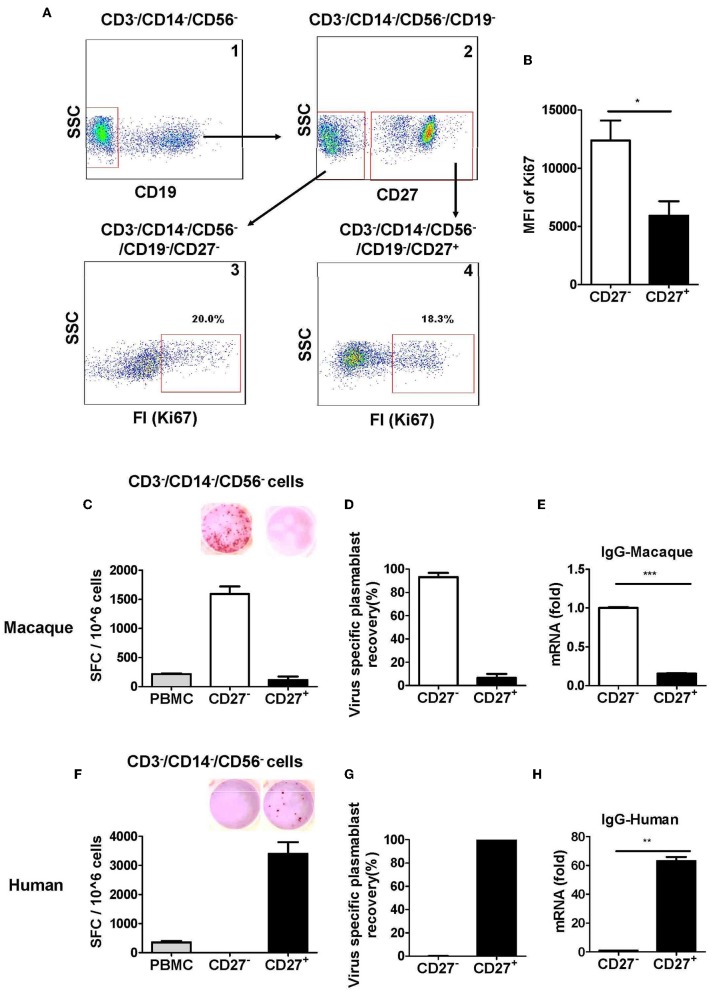
Antibody-secreting plasmablasts from Chinese rhesus macaques are primarily Ki67^+^CD19^−^CD27^−^ cells. **(A)** Flow cytometry analysis showing intracellular expression of Ki67^+^ in CD27^−^ or CD27^+^ cells. **(B)** Mean fluorescence intensity (MFI) of Ki67 expression in CD3^−^CD14^−^CD56^−^CD19^−^CD27^−^ and CD3^−^CD14^−^CD56^−^CD19^−^CD27^+^ cells subsets. PBMCs from rhesus macaques **(C,E)** (*n* = 4) and human volunteers **(F,H)** (*n* = 3) were sorted from among the CD3^−^CD14^−^CD56^−^ cells based on positive or negative CD27 expression. They were subsequently subjected to influenza A virus-specific ELISPOT assay and qRT-PCR to assess the mRNA expression levels of IgG. Data points represent the plasmablast numbers observed per million PBMCs or other cell subsets. Values represent the mean percentage of total Ig-secreting plasmablasts recovered from each cell population (Mean ± SEM). **(D)** (*n* = 4) and human volunteers **(G)** (*n* = 3). The experiment has repeated a minimum of three times. FI, fluorescence intensity; FSC, forward scatter; SSC, side scatter; SFC, spot-forming cells. *p < 0.05; **p < 0.01; ***p < 0.001.

**Figure 4:**
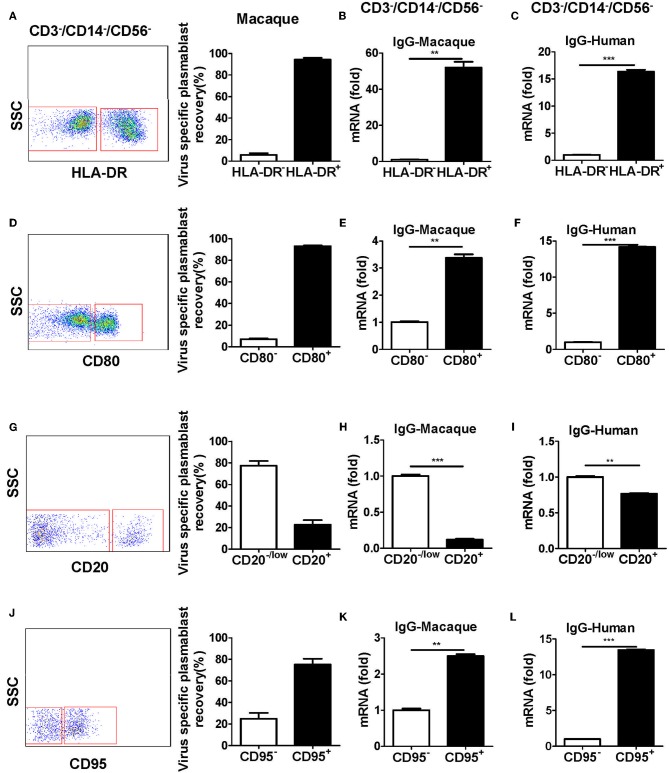
Antibody-secreting plasmablasts from Chinese rhesus macaques are CD3^−^CD14^−^CD56^−^CD19^−^CD27^−^CD20^−/low^CD80^+^HLA-DR^+^CD95^+^cells. PBMCs from rhesus macaques (*n* = 4) were sorted from among the CD3^−^CD14^−^CD56^−^CD19^−^ subset cells based on the positive or negative expression of HLA-DR **(A)**, CD80 **(D)**, CD20 **(G)**, or CD95 **(J)** and subsequently subjected to influenza virus-specific ELISPOT assay. Values represent the mean percentage of total Ig-secreting plasmablasts recovered from each cell population (Mean ± SEM). PBMCs from Chinese rhesus macaques (*n* = 4) and human volunteers (*n* = 3) were sorted from among the CD3^−^CD14^−^CD56^−^ cells based on the positive or negative expression of HLA-DR **(B,C)**, CD80 **(E,F)**, CD20 **(H,I)**, or CD95 **(K,L)** and qRT-PCR was used to assess the mRNA expression levels of IgG. The experiment has repeated a minimum of three times. **p < 0.01; ***p < 0.001.

It has been reported that CD27 is a marker expressed by human memory B cells and is also highly upregulated in human plasmablasts (7, 22). There was a discrepancy between two previous studies regarding CD27 expression in antibody-secreting plasmablasts from Indian rhesus macaques. One study showed that antibody-secreting plasmablasts were CD27^−^ (8); another study reported that antibody-secreting cells could be either CD27^+^ or CD27^−^ cells (9). Therefore, we analyzed the expression of the proliferation marker Ki67^+^ in CD27^+^ and CD27^−^ cells in the lineage-negative gate (CD3^−^CD14^−^CD56^−^CD19^−^) cell population. Although there was no significant difference in Ki67^+^ cells between the CD27^+^ and CD27^−^ cells (Figures 3A-3, 4), the mean fluorescence intensity (MFI) of Ki67^+^ was higher among the CD27^−^ cells than the CD27^+^ cells (Figure 3B). This observation suggested that most CD27^−^ cells were recently proliferating cells; these cells are more likely to be antibody-secreting plasmablasts.

We evaluated the secretion of antigen-specific antibodies from CD27^−^ and CD27^+^ cells that were sorted from among the cells in the lineage-negative gate (CD3^−^CD14^−^CD56^−^). The antigen-specific ELISPOT assay revealed that >90% of the influenza virus-specific antibody secretion was from CD27^−^ cells, whereas <10% was from CD27^+^ cells (Figures 3C,D). Therefore, most antigen-specific antibody-secreting cells were in the CD27^−^ cell population and not in the CD27^+^ cell population. To further confirm this observation, the expression of IgG mRNA in the CD27^+^ and CD27^−^ cells was determined by qRT-PCR. Compared to the CD27^+^ cells, the CD27^−^ cells had a higher level of IgG expression (Figure 3E). This demonstrated that after vaccination, the antigen-specific antibody-secreting plasmablasts from Chinese rhesus macaques were primarily CD27^−^, and they are phenotypically distinct from their human counterparts (Figures 3F–H).

### Plasmablasts Induced After Vaccination Were CD3^−^CD14^−^CD56^−^CD19^−^CD27^−^CD20^-/low^CD80^+^HLA-DR^+^CD95^+^

To further characterize plasmablasts from Chinese rhesus macaques, we sorted cells with positive and negative expression of several human plasmablast markers, including HLA-DR, CD80, CD20, and CD95 from CD3^−^CD14^−^CD56^−^ cells. The sorted cells were tested by ELISPOT for the secretion of influenza virus-specific antibodies. The expression of IgG mRNA in the cells was determined by qRT-PCR. Most of the antibody secretion activities were detected from HLA-DR^+^, CD80^+^, CD20^−/low^, or CD95^+^ cells (Figures 4A,D,G,J). These subsets expressed higher levels of IgG mRNA (Figures 4B,E,H,K). These results demonstrated that plasmablasts from Chinese rhesus macaques are HLA-DR^+^/CD80^+^/CD20^−low^/CD95^+^ cells, which is similar to human plasmablasts (Supplementary Figure 1B, Figures 4C,F,I,L).

**Figure 5:**
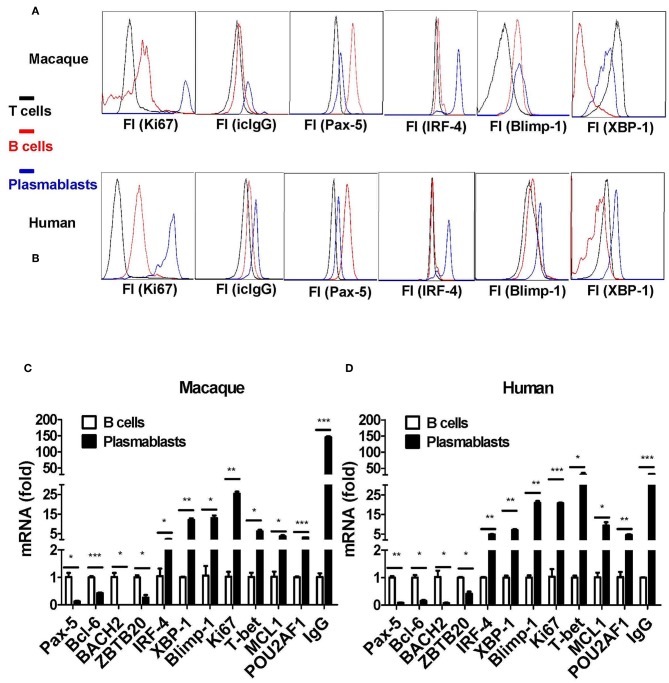
Transcriptional characterization of antibody-secreting plasmablasts from Chinese rhesus macaques. **(A)** Intracellular protein expression of Ki67, IgG, Pax-5, IRF-4, Blimp-1, and XBP-1 by plasmablasts compared with T (CD3^+^) or B (CD20^+^) cells from Chinese rhesus macaques (*n* = 4). **(B)** Intracellular protein expression of Ki67, IgG, Pax-5, IRF-4, Blimp-1, and XBP-1 by plasmablasts compared with T (CD3^+^) or B (CD20^+^) cells from human volunteers (*n* = 3). **(C)** Macaque (*n* = 4) plasmablasts and B cells (CD20^+^) were sorted at day 7 after booster vaccination. The mRNA levels of Pax-5, Bcl-6, BACH2, ZBTB20, IRF-4, XBP-1, Blimp-1, Ki67, T-bet, MCL1, POU2AF1/OBF1, and IgG were detected by qRT-PCR. **(D)** Human (*n* = 3) plasmablasts and B cells (CD20^+^) were sorted at day 7 after booster vaccination. The mRNA levels of Pax-5, Bcl-6, BACH2, ZBTB20, IRF-4, XBP-1, Blimp-1, Ki67, T-bet, MCL1, POU2AF1/OBF1, and IgG were detected by qRT-PCR. The Chinese rhesus macaque plasmablast gating strategy was as follows: CD3^−^CD14^−^CD56^−^CD19^−^CD27^−^CD20^−/low^ CD80^+^HLA-DR^+^CD95^+^. The human plasmablast gating strategy was as follows: CD3^−^CD19^+^CD20^−/low^ CD27^hi^CD38^hi^. The experiment has repeated a minimum of three times. Each bar represents Mean ± SEM. FI, fluorescence intensity; icIgG, intracellular IgG. *p < 0.05; **p < 0.01; ***p < 0.001.

Taking the results together, plasmablasts from Chinese rhesus macaques can be defined as CD3^−^CD14^−^CD56^−^CD19^−^CD27^−^CD20^−/low^CD80^+^HLA-DR^+^CD95^+^ cells. Next, we isolated these cells, as well as CD3^+^ T and CD20^+^ B cells, for comparative characterization. The CD3^−^CD14^−^CD56^−^CD19^−^CD27^−^CD20^−/low^CD80^+^HLA-DR^+^CD95^+^ cells from Chinese rhesus macaques were Ki67^+^ and intracellular IgG^+^ (Figure 5A), which is similar to human plasmablasts (Figure 5B). It is known that the fate of B cells is controlled by the expression of a network of transcription factors, including Blimp-1, IRF-4, and the spliced isoform of XBP-1 (23). These transcription factors drive the differentiation of B cells into plasmablasts and induce the expression of the molecular machinery required for antibody secretion. As assessed by intracellular staining and flow cytometry, we observed that IRF-4, XBP-1, and Blimp-1 expression levels were highest in plasmablasts relative to B and T cells (Figure 5A). Pax-5 is a transcription factor that helps B cells to maintain their identity (23). In both macaques and humans, Pax-5 expression was lower in plasmablasts than in B cells (Figure 5B). Using qRT-PCR, we also measured the mRNA transcription level of 11 transcription factors and IgG in CD3^−^CD14^−^CD56^−^CD19^−^CD27^−^CD20^−/low^CD80^+^HLA-DR^+^CD95^+^ cells and CD20^+^ B cells. Plasmablasts from Chinese rhesus macaques expressed higher levels of IRF-4, XBP-1, Blimp-1, Ki67, T-bet, MCL1, POU2AF1/OBF1, and IgG (Figure 5C) than CD20^+^ B cells. In contrast, the expression levels of Pax-5, Bcl-6, BACH2, and ZBTB20 were lower in plasmablasts than in CD20^+^ B cells (Figure 5C). We also observed a similar mRNA transcriptional profile in human plasmablasts using the following gating strategy: CD3^−^CD19^+^CD20^−/low^ CD27^hi^CD38^hi^ (Figure 5D).

**Figure 6:**
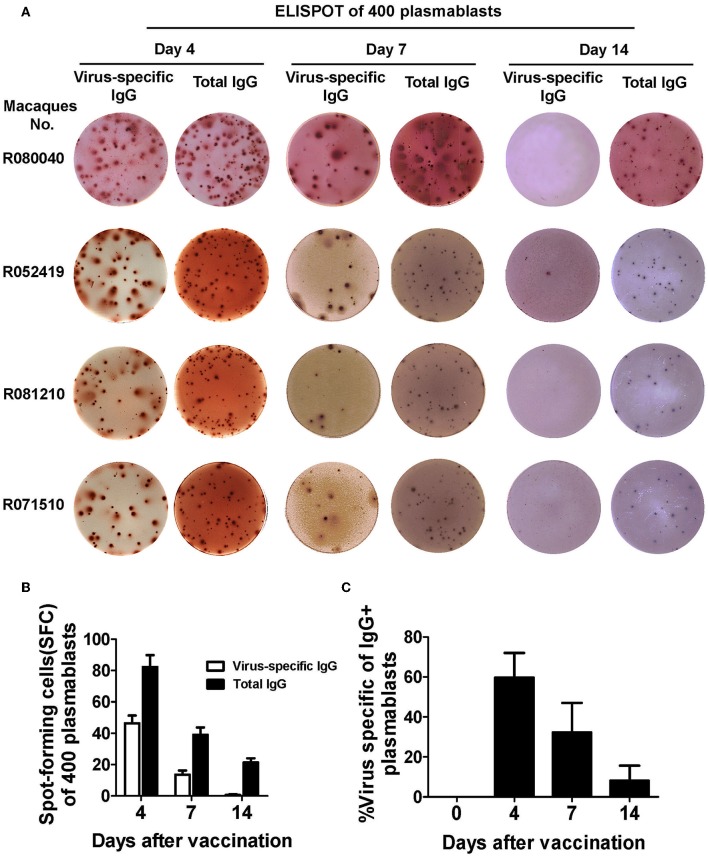
Functional confirmation of antibody-secreting plasmablasts from Chinese rhesus macaques by ELISPOT assay. **(A)** 400 sorted Chinese rhesus macaque plasmablasts were plated with inactivated influenza virus or anti-human IgG. Representative examples of the ELISPOT assay results for macaque plasmablasts at day 4, 7, and 14 after vaccination (macaque no. R080040, R052419, R081210 and R071510). **(B)** Spot-forming cells (SFC) secreting virus-specific or total IgG among 400 sorted Chinese rhesus macaque plasmablasts at day 4, 7, and 14 after vaccination (*n* = 4, Mean ± SEM). **(C)** Percentage of influenza virus-specific plasmablasts among IgG-secreting plasmablasts at indicated time points after booster vaccination. The Chinese rhesus macaque plasmablast gating strategy was as follows: CD3^−^CD14^−^CD56^−^CD19^−^CD27^−^CD20^−/low^CD80^+^HLA-DR^+^CD95^+^ (*n* = 6, Mean ± SEM). The experiment has repeated a minimum of three times.

Based on these findings, we sorted plasmablasts based on the CD3^−^CD14^−^CD56^−^CD19^−^CD27^−^CD20^−/low^CD80^+^HLA-DR^+^CD95^+^ phenotype from among the PBMCs collected at different time points after the Chinese rhesus macaques received a booster vaccination of influenza virus. We found that the magnitude of influenza virus-specific plasmablast responses was significantly higher at day 4 than at day 7 after the booster vaccination. Up to 60% of the IgG-secreting plasmablasts were influenza-specific at day 4 (Figures 6A-C). Therefore, we concluded that HLA-DR, CD80, and CD95 expression combined with a lineage-negative gate (CD3^−^CD14^−^CD56^−^CD19^−^CD27^−^CD20^−/low^) can be used to isolate plasmablasts from Chinese rhesus macaques.

### Antigen-Specific mAbs Could Be Cloned From CD3^−^CD14^−^CD56^−^CD19^−^CD27^−^CD20^-/low^CD80^+^HLA-DR^+^CD95^+^ Plasmablasts

**Figure 7:**
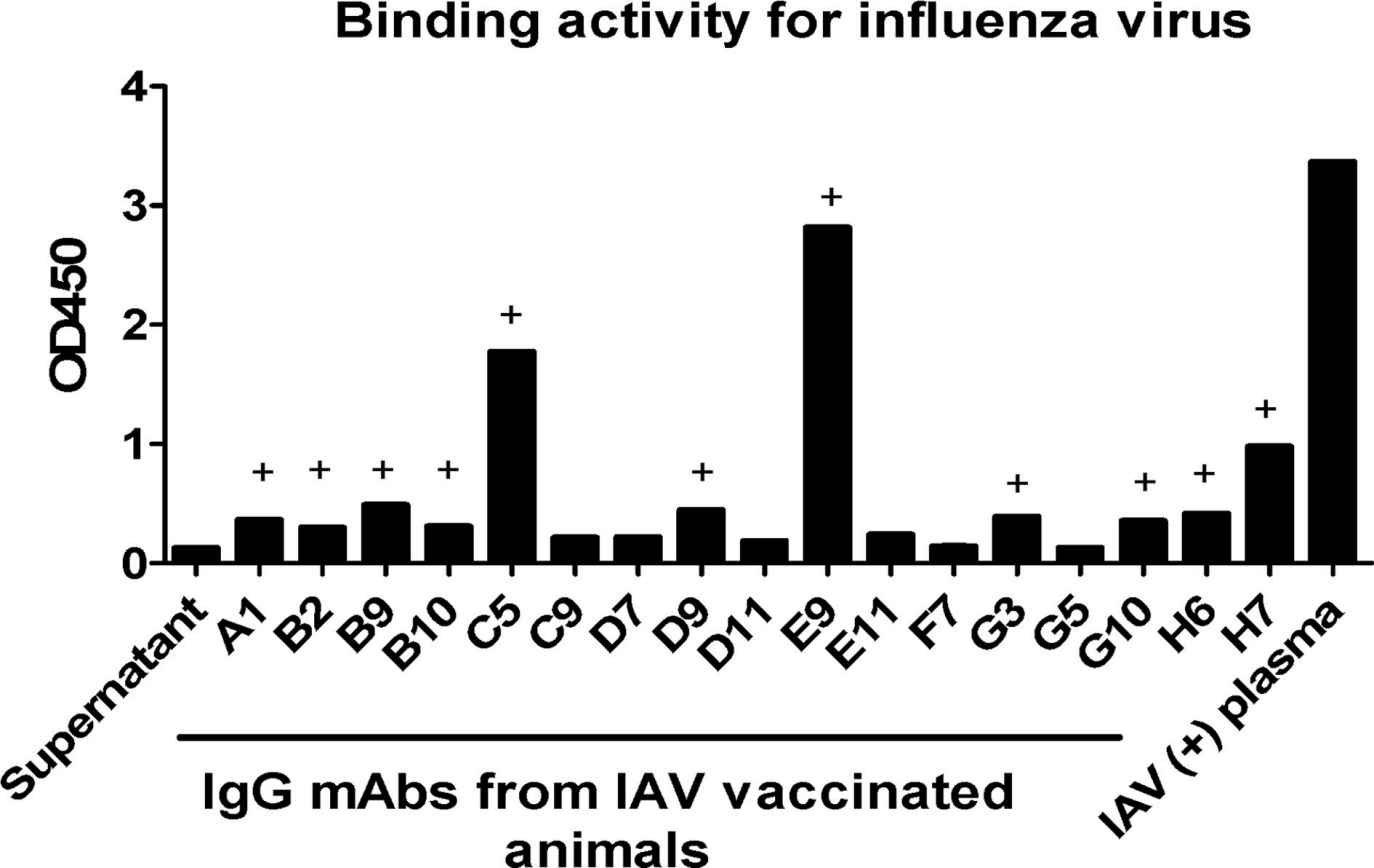
Plasmablast-derived mAbs isolated from Chinese macaques display specific reactivity to influenza viruses. Single plasmablasts from Chinese rhesus macaques were sorted into a 96-well plate at day 4 after vaccination and were used to generate cDNA. Subsequently, the cDNAs were used as templates in nested PCRs with IgG heavy and light chain-specific primers. The paired IgG heavy and light chain sequences were cloned into antibody expression vectors and expressed in 293T cells after transfection. The mAbs produced were tested by ELISA. Inactivated influenza virus was coated onto a 96-well plate at a concentration of 1 μg/ml. At 72 h post transfection, 100 μl IgG expression supernatant was used as the primary antibody. HRP-conjugated anti-human antibody was used as the detection antibody. Influenza A virus (IAV)-positive plasma was used as a positive control. The OD450 values of the blank control were subtracted from those of the other wells. (+) The minimum mAb concentration indicating antigen-specific binding was defined as an OD value ≥2-fold the OD value of the negative control.

One important application of plasmablast identification is the use of single-cell PCR to clone heavy and light chain-paired mAbs from single plasmablasts. To demonstrate this utility, at day 4 after booster influenza virus vaccination, we sorted CD3^−^CD14^−^CD56^−^CD19^−^CD27^−^CD20^−/low^CD80^+^HLA-DR^+^CD95^+^ plasmablasts into one 96-well plate, with one cell per well. The single cells were subjected to nested PCR with Ig heavy and light chain specific primers, as described previously (20) (Supplementary Tables 3, 4). Overall, either Ig heavy or light chain amplicons could be obtained from 86 out of the 96 isolated plasmablasts, confirming that these cells expressed antibodies. We sequenced 17 mAbs with both Ig heavy and light chains successfully amplified from the same cell (Supplementary Table 5). An expression plasmid for each heavy and light chain pair was constructed and transfected into 293T cells to express full-length IgG1 antibodies, as previously described (2). After transfection, antibodies secreted into the culture medium were used for ELISA using influenza virus (H3N2/Switzerland/2013) as the antigen. Eleven out of the 17 mAbs (64.7%) showed positive reactivity to influenza virus H3N2/Switzerland/2013 (Figure 7), confirming that cells sorted based on the CD3^−^CD14^−^CD56^−^CD19^−^CD27^−^CD20^−/low^CD80^+^HLA-DR^+^CD95^+^ phenotype are indeed antibody-secreting plasmablasts. We also confirmed the binding of some mAbs, including mAbs H7, B9, and B10. A previously published human broad-spectrum anti-influenza virus mAb MEDI8852 was used for comparison (24). These mAbs were expressed in 293 cells and were purified using protein A agarose. mAb H7 exhibited higher affinity with saturation of binding achieved at low IgG concentrations. mAbs B9 and B10 showed comparable binding to H3N2 infected cells as mAb MEDI8852 (Supplementary Figure 2). We also estimated the affinity constants (Ka) of these mAbs from FACS data. When the binding rate of H3N2 infected cells to antibody increased to 50%, the reciprocal of corresponding antibody concentration is the Ka of that antibody, as shown in Table 1.

**Table 1:** Affinity constant (Ka) measurements of mAbs by FACS.

**mAbs**	**Ka (L/Mol)**
MEDI8852	2.02 × 10^8^
H7	2.68 × 10^8^
B9	1.31 × 10^9^
B10	6.97 × 10^8^

## Discussion

NHPs, especially rhesus macaques, are regarded as the most human-relevant animal models for evaluating experimental vaccines, pathogen infections and immune responses (1, 3, 6, 25–27). Compared to other animal species (apart from chimpanzees), the immunoglobulin gene of rhesus macaques shares the highest degree of homology with that of humans (28, 29). Rhesus macaques have been exploited as a valuable source for cloning therapeutic mAbs with a high degree of similarity to human antibodies (2, 4, 5, 8, 9). Although transgenic mice carrying human immunoglobulin genes have been developed and used to generate humanized antibodies, most of these mice are commercially protected and maybe not accessible. We previously reported that mAbs obtained from Chinese rhesus macaques could achieve >97% homology with human mAbs (2). Antibody-secreting plasmablasts have been used as a source to clone antigen-specific mAbs from humans and rhesus macaques (7–10). However, little information is available about the phenotypic characterization and kinetics of plasmablasts from Chinese rhesus macaques after infection or vaccination. In this study, we used influenza virus as a model antigen for defining the phenotypic markers of plasmablasts from Chinese rhesus macaques and for verifying the cloning of antigen-specific mAbs.

It has been well-accepted that human plasmablasts can be defined as CD3^−^CD19^+^CD20^−/low^CD27^hi^CD38^hi^ cells. However, these cell surface markers cannot simply be applied to identify plasmablasts from Chinese rhesus macaques (Figure 1). Although human and rhesus macaques genome are 95.3% identical (26), some proteins may have variations in amino acid sequences. Therefore, an anti-human mAb that recognizes a particular epitope on a human protein may not find the same epitope on macaque cells. We thus firstly assessed the cross-reactivity of a variety of anti-human antibodies to ensure that they also recognize the same proteins in Chinese rhesus macaques (Supplementary Table 1). Finally, we defined the phenotype of antibody-secreting plasmablasts from Chinese rhesus macaques as CD3^−^CD14^−^CD56^−^CD19^−^CD27^−^CD20^−/low^CD80^+^HLA-DR^+^CD95^+^.

We observed that HLA-DR is expressed on Chinese macaque plasmablasts, as previously described for their human and Indian macaque counterparts (7, 8, 13). CD80, also known as B7.1, provides costimulatory signals to T cells and is a typical marker of B cell activation. CD80 is upregulated on activated B cells, and this marker has been described as a regulator of germinal center development as well as antibody-secreting cells in mice (30), and it is expressed on human and Chinese and Indian macaque plasmablasts. CD95, also known as death receptor Fas, has been identified as a key regulator of activation-induced B-cell death. CD95 is highly expressed on activated B cells (31). We found that CD95 was expressed on both human and Chinese macaque plasmablasts. The most striking differences between humans and Chinese rhesus macaques were observed for CD19 and CD27 in our study. While human plasmablasts expressed high levels of CD19 and CD27, the macaque plasmablasts were low or negative for both markers (Supplementary Table 6).

In this study, the expression pattern of a series of transcription factors between human and macaque human plasmablasts were compared to further confirm the identity of Chinese rhesus macaque plasmablasts isolated based on the markers we proposed. Among these transcription factors, Blimp-1 is a key factor that can drive the maturation of B cells into antibody-secreting cells (32, 33). Recently, POU2AF1/OBF1 was found to act upstream of Blimp-1 (34). Moreover, the maintenance of MCL1 expression in antibody-secreting cells is crucial for their survival, and the survival pathway is independent of the Blimp-1-dependent component of antibody-secreting cell differentiation (35). POU2AF1/OBF1 and MCL1 were found to be highly expressed on vaccination-induced plasmablasts from Chinese rhesus macaques. These two factors may play important roles in triggering plasmablast differentiation and supporting cell survival.

Importantly, plasmablasts isolated from Chinese rhesus macaques were confirmed functionally. Sorted plasmablasts were tested using antigen-specific ELISPOT assays to detect the secretion of antibodies that react to influenza virus. In this study, we focused on IgG-producing plasmablasts. Most human plasmablasts have been shown to be IgG-secreting cells, but there are minor quantities of IgA- and IgM-secreting cells at 7 days after vaccination with influenza virus (7). A significant number but not all cells were positive for IgG production (Figures 6A,B). Some cells may not produce antibodies due to damages during isolation, which could account for 30–50% of FACS-sorted cells (8). Among the plasmablasts that produced a detectable amount of IgG, most were antigen-specific (Figures 6A-C). Therefore, antigen-specific mAbs can be cloned directly at a high success rate from freshly sorted plasmablasts. On the other hand, plasmablasts can be cultured for 1–2 days followed by antigen-specific ELISA to identify single plasmablast that produces antigen-specific antibodies. However, single-cell culture of plasmablasts is a delicate process which demands to maintain high cell viability and antibody secretion. More antigen proteins may be consumed to perform ELISA screen.

In this study, we demonstrated that among the 17 mAbs cloned from sorted plasmablasts, 11 mAbs were specific for influenza virus. Another approach to clone antigen-specific mAbs is to use fluorescence-labeled antigens as probes to isolate antigen-specific memory B cells (4, 36, 37). However, when the binding epitopes of an antigen are not defined or available, cloning mAbs directly from plasmablasts after vaccination would have a significant advantage. This allows characterization of B cell responses and cloning of mAbs based on plasmablasts from peripheral blood after vaccination or infection (7, 10, 11). The magnitude of the plasmablast response has been shown to correlate directly with neutralizing antibody titers and the potential to clear the infection. In humans, up to 70% of plasmablasts are antigen-specific at the peak of their response at 7 days after booster vaccination. Therefore, antigen-specific plasmablasts are excellent sources for cloning antigen-specific mAbs (7, 12). Earlier studies in Indian rhesus macaque showed that antibody-secreting cells peaks at either 4 and 7 days after the booster vaccination, but these two studies used a different set of markers (8). In our study, we found that up to 60% of IgG-secreting plasmablasts from Chinese rhesus macaques were antigen-specific at day 4, the magnitude of influenza virus-specific plasmablast responses was higher at day 4 than at day 7 after the booster vaccination (Figures 6A–C). It is possible that the peak of plasmablast response may vary due to antigen or adjuvant used, as well as the route of vaccination.

Influenza virus-specific memory B cells can be rapidly reactivated to yield plasmablasts after booster vaccination. Kinetic analysis of plasmablast responses is important for the design and appropriate timing of sample collection in vaccine and antibody studies. In humans, the number of plasmablasts in the peripheral blood peaks at day 7 after booster vaccination with influenza virus (7, 12). The rapid accumulation of plasmablasts suggests that these cells are the result of rapid clonal expansion. Somatic hypermutations in the immunoglobulin genes lead to the generation of high-affinity antibodies. Antigen-specific plasmablasts accumulate more somatic mutations than other B cell populations in humans (7). In this study, we found that plasmablasts exhibited a robust response at day 4 after booster vaccination in Chinese rhesus macaques, and this response occurred earlier than in humans after booster vaccination, also with influenza virus. Therefore, Chinese rhesus macaque plasmablasts may have a faster clonal expansion after vaccination. In the future, it would be desirable to analyze antibody repertoires at different days after vaccination to understand more details in antibody clonal expansion and somatic hypermutation.

In summary, we defined the phenotypic markers for isolating antibody-secreting plasmablasts from Chinese rhesus macaques. Using this phenotype definition, single plasmablasts could be sorted using flow cytometry. mAbs with paired Ig heavy and light chain gene sequences could be cloned into expression plasmids using single-cell PCR for further analysis. This study should facilitate the evaluation of vaccination-induced plasmablast response and the efficient cloning of antigen-specific mAbs from Chinese rhesus macaque plasmablasts.

## Data Availability Statement

The raw data supporting the conclusions of this manuscript will be made available by the authors, without undue reservation, to any qualified researcher.

## Ethics Statement

This study was carried out in accordance with the recommendations of Human Research Ethics Review Committee of Guangzhou Institute of Biomedicine and Health with written informed consent from all subjects. All subjects gave written informed consent in accordance with the Declaration of Helsinki. The protocol was approved by the Human Research Ethics Review Committee of Guangzhou Institute of Biomedicine and Health. This study was carried out in accordance with the recommendations the guidelines of the NIH Guide for the Care and Use of Laboratory Animals and the policies and procedures of Guangzhou Institute of Biomedicine and Health and the protocol was approved by the Institutional Animal Care and Use Committee of Guangzhou Institute of Biomedicine and Health.

## Author Contributions

LC and PL conceived and designed the experiments. PL, FZ, LW, JLi, JLu, YF, YY, PH, WF, and RL performed the experiments. PL, FZ, ZZ, and XN analyzed the data. WP, CL, and HY contributed reagents and materials. PL, FZ, YT, XN, and LC wrote the paper. All authors commented on the manuscript.

### Conflict of Interest

The authors declare that the research was conducted in the absence of any commercial or financial relationships that could be construed as a potential conflict of interest.
