# Supplementary material for: Phenotypic Characterization of Chinese Rhesus Macaque Plasmablasts for Cloning Antigen-Specific Monoclonal Antibodies
Source: Front Immunol. 2019 Oct 11;10:2426. doi: 10.3389/fimmu.2019.02426 (PMC6798180; doi:10.3389/fimmu.2019.02426)
Supplement: Supplementary file 1 [file Data_Sheet_1.docx]

**Supplementary Tables**

**Supplementary Table 1. Antibodies used for analytical flow cytometry and / or cell sorting**

| **Antigen** | **Clone** | **Fluorophore** | **Supplier** | **Staining** | **Reactivity** |
| --- | --- | --- | --- | --- | --- |
| CD3 | SP34-2 | Pacific Blue | BD Biosciences | Surface | Human |
| CD3 | SP34-2 | PE | BD Biosciences | Surface | Human |
| CD14 | M5E2 | PE | BD Biosciences | Surface | Human |
| CD14 | M5E2 | Biotin | Biolegend | Surface | Human |
| CD11c | 3.9 | PE | eBioscience | Surface | Human |
| CD56 | B159 | PE | BD Biosciences | Surface | Human |
| CD56 | HCD56 | Biotin | Biolegend | Surface | Human |
| CD19 | J3-119 | PE-Cy7 | Beckman Coulter | Surface | Human |
| CD19 | J3-119 | Pacific Blue | Beckman Coulter | Surface | Human |
| CD20 | 2H7 | PE-CF594 | BD Biosciences | Surface | Human |
| CD20 | 2H7 | PE | BD Biosciences | Surface | Human |
| CD27 | CLB-27/1 | PE | Invitrogen | Surface | Human |
| CD27 | M-T271 | APC | BD Biosciences | Surface | Human |
| CD27 | M-T271 | PerCP-Cy5.5 | BD Biosciences | Surface | Human |
| CD38 | AT-1 | FITC | Stemcell Technologies | Surface | Human |
| CD80 | L307.4 | Alexa Fluor 700 | BD Biosciences | Surface | Human |
| CD80 | L307.4 | PE-Cy5 | BD Biosciences | Surface | Human |
| CD80 | L307.4 | APC-Cy7 | BD Biosciences | Surface | Human |
| CD95 | DX2 | PE | BD Biosciences | Surface | Human |
| CD95 | DX2 | FITC | BD Biosciences | Surface | Human |
| CD95 | DX2 | PE-Cy5 | BD Biosciences | Surface | Human |
| CD123 | 7G3 | FITC | BD Biosciences | Surface | Human |
| HLA-DR | G46-6 | APC | BD Biosciences | Surface | Human |
| IgG | G18-145 | Biotin | BD Biosciences | Surface | Human |
| IgG | G18-145 | APC-H7 | BD Biosciences | Surface/  Intracellular | Human |
| IgG | G18-145 | PE-CF594 | BD Biosciences | Surface/  Intracellular | Human |
| Ki67 | B56 | PE | BD Biosciences | Intracellular | Human |
| Ki67 | B56 | Alexa Fluor 647 | BD Biosciences | Intracellular | Human |
| Ki67 | B56 | PE-Cy7 | BD Biosciences | Intracellular | Human |
| Pax-5 | 1H9 | Alexa Fluor 647 | BD Biosciences | Intracellular | Human/Mouse |
| IRF-4 | 3E4 | FITC | eBioscience | Intracellular | Human/Mouse |
| XBP-1 | Q3-695 | Alexa Fluor 647 | BD Biosciences | Intracellular | Human/Mouse |
| Bcl-6 | K112-91 | Alexa Fluor 647 | BD Biosciences | Intracellular | Human/Mouse |
| Blimp-1 | 6D3 | PE | BD Biosciences | Intracellular | Human/Mouse |
| Streptavidin | — | PE | BD Biosciences | Surface | Human/Mouse |
| Streptavidin | — | FITC | BD Biosciences | Surface | Human/Mouse |
| Streptavidin | — | V500 | BD Biosciences | Surface | Human/Mouse |
| Streptavidin | — | APC | BD Biosciences | Surface | Human/Mouse |
| Isotype IgG1, κ | MOPC-21 | PE/FITC | BD Biosciences | Surface/  Intracellular | Mouse |

**Supplementary Table 2. qPCR primers**

| **Target gene** | **Primer sequence ( 5’ to 3’ )** |
| --- | --- |
| Monkey/Human |  |
| β-actin | Forward：GCATTCTCACCCTGAAGTA |
|  | Reverse：GCAGCTCATTGTAGAAGGT |
| Pax-5 | Forward：TCCGCCAGAGGATAGTGGAA |
|  | Reverse：CCAGGCTTGATGCTTCCTGT |
| Bcl-6 | Forward：AAGGCCAGTGAAGCAGAGAT |
|  | Reverse：TCAGTGGCAGGTTGTTCTCC |
| BACH-2 | Forward：CGTTCTCGGAAGCAGACAGTG |
|  | Reverse：CCGTCGGACATCATGAATAAACTC |
| ZBTB20 | Forward：ATGCTAGAACGGAAGAAACCCA |
|  | Reverse：TGTGAGCGTGAGAGTTTGTC |
| IRF-4 | Forward：AGCTTGTGAAAATGGTTGCC |
|  | Reverse：GCAGTCTGAGAG/ACGCCAAG |
| XBP-1 | Forward：CTGAGTCCGCAGCAGGTG |
|  | Reverse：GTCCAGAATGCCCAACAGG |
| Blimp-1 | Forward：TCTCCAATCTGAAGGTCCACC |
|  | Reverse：TGGCAGACCTGGCATTCAT |
| Ki67 | Forward：TGCAGCTGATCCCATTTCC |
|  | Reverse：GCTATTGTCAAGACATTGCG |
| T-bet | Forward：CCCCTTGGTGTGGACTGAGA |
|  | Reverse：ACGCGCCTCCTCTTAGAGTC |
| MCL1 | Forward：CAGAGGAGGAGGAGGACGA |
|  | Reverse：CATCCTTGGAAGGCCGTCT |
| POU2AF1 / OBF1 | Forward：CCAATGTCACGACAAGAAGC |
|  | Reverse：GTACTGCAGGGTGGAGGTG |
| IgG | Forward：ACTCCGACGGCTCCTTCTTC |
|  | Reverse：CTTCTGCGTGTAGTGGTTGTGC |

**Supplementary Table 3. First round PCR primer list**

|  | **Primer** | **Sequence (5’ – 3’)** |
| --- | --- | --- |
| 1^st^ round heavy chain primer | VH1a-1^st^ | TGGCAGCAGCTACAGGYGC |
|  | VH1b-1^st^ | TGACAGCAGCCACAGGTGC |
|  | VH1c-1^st^ | TGGCAGCAGCTACAGGYGG |
|  | VH2-1^st^ | GTCCCGTCCTGGGTCTTGTC |
|  | VH3-1^st^ | AGGTGTCCAGTGTGAGGTGC |
|  | VH4-1^st^ | ATGAAGCACCTGTGGTTC |
|  | VH5-1^st^ | ATGGGGTCAACTGCCATC |
|  | VH6-1^st^ | ATGTCTGTCTCCTTCCTCA |
|  | VH7-1^st^ | GCAGCAACAGGTGCCCACTC |
|  | IgG-1^st^ | GTTCAGGGAAGTAGTCCTTGAC |
| 1^st^ round kappa chain primer | VK1a-1^st^ | ATGGACATGAGGGTCCCCGC |
|  | VK1b-1^st^ | GGCTCCTKCTGCTCTGGCTC |
|  | VK2-1^st^ | ATGRGYTCCCTGCTCAR |
|  | VK3-1^st^ | ATGGAARCCCCAGCWCAGC |
|  | VK4/5-1^st^ | CTSTTSCTYTGGATCTCTG |
|  | VK6/7-1^st^ | CTSCTGCTCTGGGYTCC |
|  | Kappa-1^st^ | GTCCTGCTCTGTGACACTCTC |
| 1^st^ round lambda chain primer | VL1-1^st^ | ATGGCCTGGTYYCCTCTC |
|  | VL2/7/10-1^st^ | ATGGCCTGGRCTCTGCTCC |
|  | VL3a-1^st^ | ATGGCCTGGATTCCTCTC |
|  | VL3b-1^st^ | ATGGCCTGGACCTTTCTC |
|  | VL3c-1^st^ | ATGGCCTGGACCCCTCCC |
|  | VL4a-1^st^ | ATGGCCTGGGTCTCCTTC |
|  | VL4b-1^st^ | ATGGCCTGGACCCCACTC |
|  | VL5/11-1^st^ | ATGGCCTGGACTCCTCTC |
|  | VL6-1^st^ | ATGGCCTGGGCTCCACTCC |
|  | VL8-1^st^ | ATGGCCTGGATGATGCTTC |
|  | VL9-1^st^ | ATGGCCTGGGCTCCTCTG |
|  | Lambda-1^st^ | TGTTGCTCTGTTTGGAGGG |

Y: C+T; K: G+T; R: A+G; W: A+T; S: C+G.

**Supplementary Table 4. Second round PCR primer list**

|  | **Primer** | **Sequence (5’ – 3’)** |
| --- | --- | --- |
| 2^nd^ round heavy chain primer | VH1a-2^nd^ | CAGGTSCAGCTGGTGCAGTCYGGG |
|  | VH1b-2^nd^ | GAGGTCCAGCTGGTRCAGTCTGGG |
|  | HV2-2^nd^ | CAGGTSACCTTGAAGGAGTCTG |
|  | HV3-2^nd^ | GAGGTGCAGCTGGTGGAGTCTGG |
|  | VH4a-2^nd^ | CAGSTGCAGCTGCAGGAGTCGG |
|  | VH4b-2^nd^ | CAGGTGCAGCTGCAGCAGTGGG |
|  | VH5/7-2^nd^ | GAGGTGCAGCTGGTGCARTCTGG |
|  | VH6-2^nd^ | CAGGTGCAGCTGCAGGAGTCAGG |
|  | IgG-2^nd^ | GCCAGGGGGAAGACCGATG |
| 2^nd^ round kappa chain primer | VK1-2^nd^ | GACATYCAGATGACSCAGTCTCC |
|  | VK2a-2^nd^ | GACATYCAGATGACYCAGTCTCC |
|  | VK2b-2^nd^ | GATRTTGTGATGACYCAGWCTC |
|  | VK3a-2^nd^ | TGACGCAGTCTCCAGCCACCCTG |
|  | VK3b-2^nd^ | CAAGTTATATTGACWCAGTCTCC |
|  | VK3c-2^nd^ | TGACGCAGTCYCCAGCCACC |
|  | VK3d-2^nd^ | TGACACAATCTCCAGCCACCTTG |
|  | VK4-2^nd^ | GACATTGTGATGACCCAGTCTC |
|  | VK5-2^nd^ | GAAACGACACTCACGCAGTCTC |
|  | VK6-2^nd^ | GAWATTGTGMTGACTCAGTCTC |
|  | VK7-2^nd^ | GACATTGTGCTGACCCAGTCTC |
|  | Kappa-2^nd^ | ATTCAGCAGGCACACAACAGAG |
| 2^nd^ round lambda chain primer | VL1a-2^nd^ | CAGTCTGTGCTGACTCAACC |
|  | VL1b-2^nd^ | CAGTCTGTGCTGACRCAGCC |
|  | VL1c/5b-2^nd^ | CAGTCTGTGCTGACTCAGCC |
|  | VL2a-2^nd^ | CAGGCTGCCCTGACTCAGYC |
|  | VL2b-2^nd^ | CAGGCTGCCCCGACTCAGCC |
|  | VL3-2^nd^ | TCCTATGAGCTGACWCAGCCAC |
|  | VL4a-2^nd^ | CAGCCTGTGCTGACTCAGTC |
|  | VL4b-2^nd^ | CTGCCTGTGCTGACTCAGC |
|  | VL5a-2^nd^ | CAGCCTGTGCTGACCCAGCC |
|  | VL6-2^nd^ | GAGGTTGTGTTCACTCAGCCCCATT |
|  | VL7-2^nd^ | CAGGCTGTAGTGACTCAGGAG |
|  | VL8-2^nd^ | GAGACTGTGGTGACCCAGGAG |
|  | VL9/11-2^nd^ | CAGCCTGTGCTGACTCAGCC |
|  | VL10-2^nd^ | CAGGCAGGGCTGACTCAG |
|  | Lambda-2^nd^ | AGACACACTAGTGTGGCCTTG |

S: C+G; Y: C+T; R: A+G; W: A+T; M: A+C.

**Supplementary Table 5. Antibody sequence analysis**

| **mAbs** | **VH family** | **DH family** | **JH family** | **CDR3 sequence(H)** | **VK(L) family** | **JK(L) family** | **CDR3 sequence(K/L)** |
| --- | --- | --- | --- | --- | --- | --- | --- |
| A1 | IGHV4-2*01 | IGHD1-8*01 | IGHJ4*01 | ATSDGGSYRDLFDL | IGLV3S12*01 | IGLJ2*01 | DPWDNSAYQA |
| B2 | IGHV4-2*01 | IGHD1-8*01 | IGHJ4*01 | ARVCGRFLD | IGKV3S11*01 | IGKJ2*01 | QQYNNWNRFYS |
| B9 | IGHV4-2*01 | IGHD2-2*01 | IGHJ1*01 | ARGGDSGAPYDFEF | IGLV1-15*01 | IGLJ3*01 | VVWDDSLSGVF |
| B10 | IGHV1-1*01 | IGHD5-3*01 | IGHJ4*01 | AVGGGGGYNYFDS | IGKV2S10*01 | IGKJ2*01 | LQTLIFPYT |
| C5 | IGHV4-2*01 | IGHD2-6*01 | IGHJ4*01 | TRKAGYCGGGVCPTFDY | IGKV1S6*01 | IGKJ2*01 | QQGYLAPYT |
| C9 | IGHV5-2*01 | IGHD3-1*01 | IGHJ4*01 | AEGYYGSGEPFDH | IGLV1S1*01 | IGLJ6*01 | QTYDSRLTADV |
| D7 | IGHV4-2*01 | IGHD4-2*01 | IGHJ4*01 | ARQSTFVDL | IGLV3-6*01 | IGLJ2*01 | QVWDTTTIL |
| D9 | IGHV4-2*01 | IGHD2-1*01 | IGHJ1*01 | AGMYSYYSGPLES | IGLV3-5*01 | IGLJ1*01 | DSWDSTDSRYI |
| D11 | IGHV4-2*01 | IGHD1-1*01 | IGHJ4*01 | ARDSAVGTMGFDY | IGKV1S6*01 | IGKJ2*01 | HQTYNTPYS |
| E9 | IGHV4-2*01 | IGHD1-7*01 | IGHJ4*01 | VGSKVWFFDY | IGLV3-6*0 | IGLJ6*01 | QVWDSDIKYV |
| E11 | IGHV4-2*01 | IGHD6-3*01 | IGHJ4*01 | ARVFSGIWTYFDY | IGLV3-4*01 | IGLJ2*01 | QVWDSSRDLL |
| F7 | IGHV4-2*01 | IGHD6-2*01 | IGHJ4*01 | AAIMATGPLIFDF | IGLV3-1*01 | IGLJ6*01 | QVWDSNSDHPV |
| G3 | IGHV4-2*01 | IGHD5-1*01 | IGHJ5-1*01 | RPLTATLKRGGWFDV | IGLV1-8*01 | IGLJ6*01 | QTWDSGLNGEV |
| G5 | IGHV3-13*01 | IGHD2-2*01 | IGHJ1*01 | TTDFYCSGSACYGEYFQF | IGLV6-2*01 | IGLJ3*01 | QSAVGRFTLL |
| G10 | IGHV4-2*01 | IGHD2-4*01 | IGHJ4*01 | VRADYSGSYFEYYFDF | IGLV3-1*01 | IGLJ2*01 | QVGASSSDRPL |
| H6 | IGHV4-2*01 | IGHD3-3*01 | IGHJ3*01 | ARNNVWTPYLGWAAFDF | IGLV2S2*01 | IGLJ1*01 | NSYAGGNTFI |
| H7 | IGHV1-1*01 | IGHD2-3*01 | IGHJ4*01 | VRGGVTEVTAPMFDY | IGLV1S6*01 | IGLJ2*01 | STWDNNLNAWI |

**Supplementary Table 6. Markers of vaccine-induced plasmablasts in PBMCs of humans and Chinese rhesus macaques**

| **Marker** | **Humans** | **Chinese rhesus macaques** |
| --- | --- | --- |
| CD3 | – | – |
| CD14 | – | – |
| CD19 | + | – |
| CD20 | –/low | –/low |
| CD27 | ++ | – |
| CD56 | – | – |
| CD80 | + | + |
| CD95 | + | + |
| HLA-DR | + | + |
| Ki67 | ++ | ++ |
| icIgG | + | + |

**Supplementary Figure legends**

**Supplementary Fig. 1. Analysis of the plasmablasts induced by influenza vaccination in humans.** PBMCs obtained at 7 days after vaccination in human (n=4). As for flow cytometry, the PBMCs were surface stained with a cocktail of fluorescein labeled antibodies, washed and resuspended in buffer. Intracellular staining was done after surface staining. The cells were permeabilized and underwent intracellular staining. The stained cells were run on a BD LSRFortessa cell analyzer. (**A**) Intracellular expression of Ki67 by plasmablasts compared with T (CD3^+^) or B (CD20^+^) cells. (**B**) Surface expression of HLA-DR, CD80, CD20 and CD95 by plasmablasts compared with T (CD3^+^) or B (CD20^+^) cells. The experiment was repeated a minimum of three times. FI: fluorescence intensity.

**Supplementary Fig. 2. FACS titration of mAb binding to influenza virus infected cells.** The binding of mAbs were measured by flow cytometry. MDCK cells were infected with influenza virus A/H3N2/Switzerland/2013. Purified mAbs H7, B9, B10 were used as primary antibody at different concentration (0, 0.25, 0.5, 1.0, 2.0, and 4.0 μg/ml). A previously published broad-spectrum anti-influenza virus mAb MEDI8852 was used for comparison, APC-H7 conjugated anti-human antibody was used as secondary antibody. Bound IgG was detected in FACS. The Y-axis represents the mean fluorescence intensity (MFI) while the X-axis represents the concentration of mAbs. The experiment was repeated three times.
