# Supplementary figures and images for: Phenotypic Characterization of Chinese Rhesus Macaque Plasmablasts for Cloning Antigen-Specific Monoclonal Antibodies
Source: Front Immunol. 2019 Oct 11;10:2426. doi: 10.3389/fimmu.2019.02426 (PMC6798180; doi:10.3389/fimmu.2019.02426)

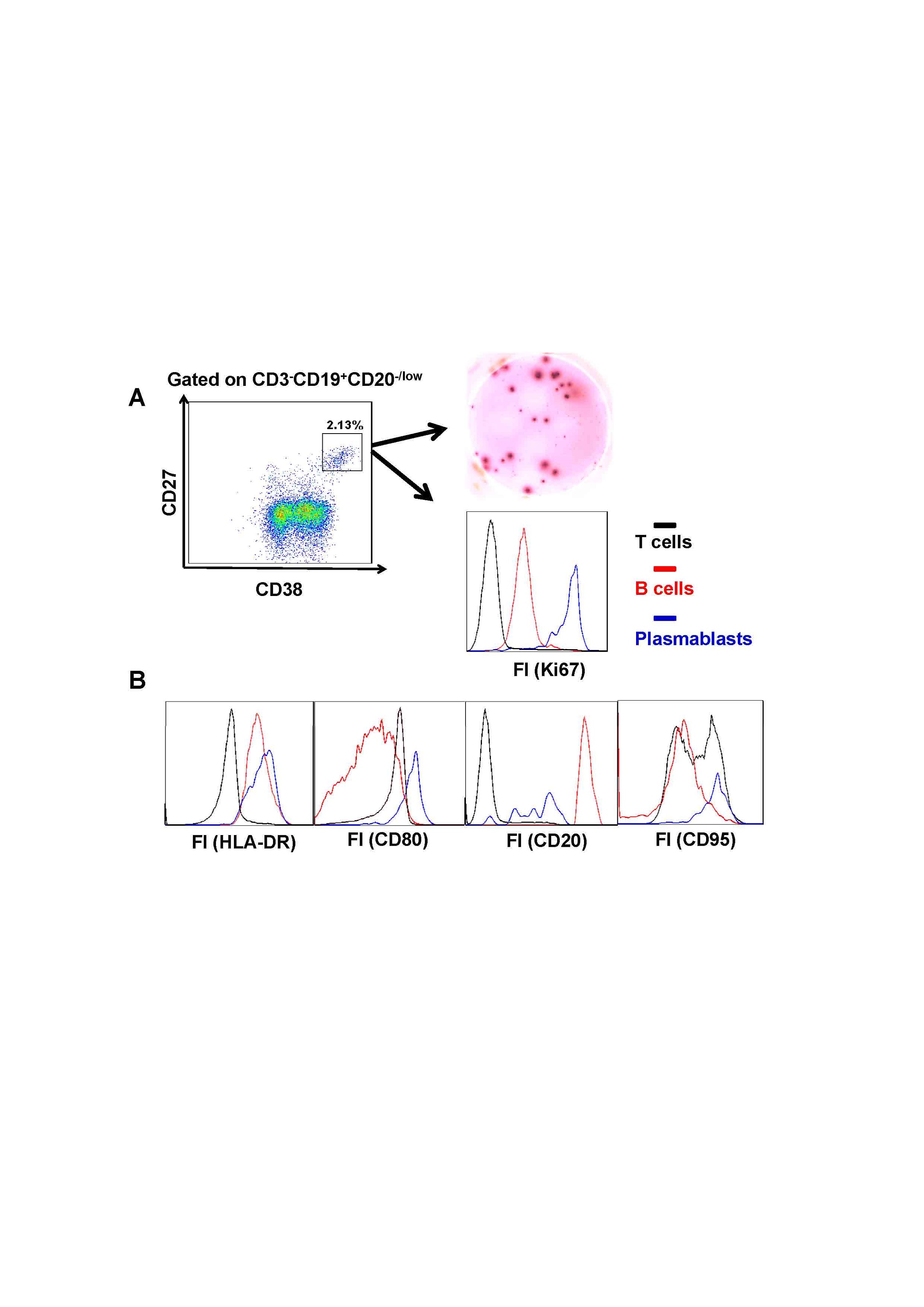

Supplement: Supplementary file 2 [file Image_1.TIFF]

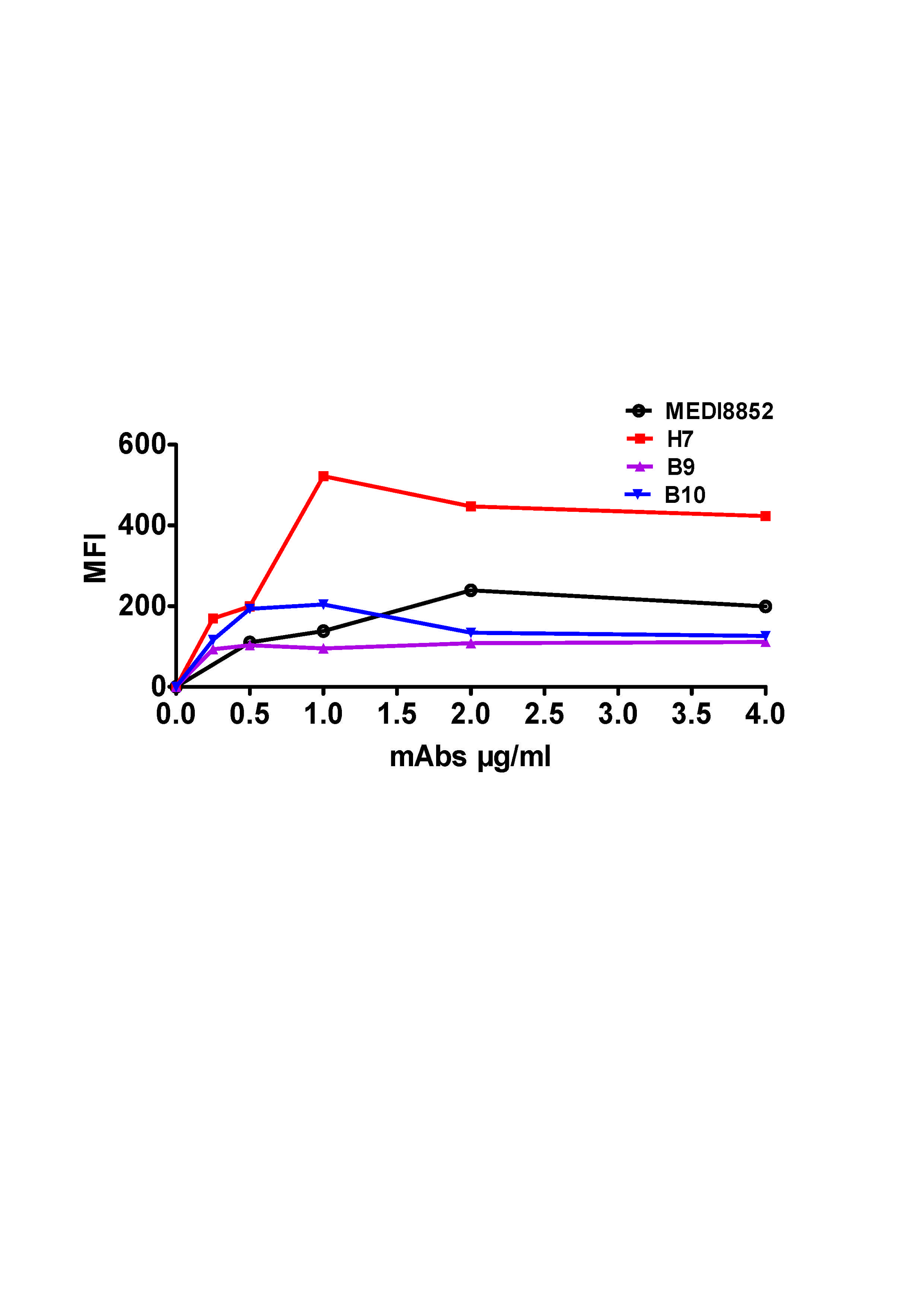

Supplement: Supplementary file 3 [file Image_2.TIFF]
